# Association Between Early Posttransplant Graft Perfusion and CLAD‐Free Survival After Single Lung Transplantation

**DOI:** 10.1155/joot/7647492

**Published:** 2026-10-15

**Authors:** Shin Tanaka, Megumi Ishihara, Yujiro Kubo, Haruchika Yamamoto, Kumi Nakajima, Hidejiro Torigoe, Shuji Okahara, Kazuhiko Shien, Ken Suzawa, Kentaroh Miyoshi, Mikio Okazaki, Seiichiro Sugimoto, Shinichi Toyooka

**Affiliations:** ^1^ Department of General Thoracic Breast, and Endocrinological Surgery Okayama University Graduate School of Medicine, Dentistry and Pharmaceutical Sciences Okayama Japan okayama-u.ac.jp; ^2^ Department of General Thoracic Surgery and Organ Transplant Center Okayama University Hospital Okayama Japan okayama-u.ac.jp; ^3^ Department of Thoracic Surgery and Lung Transplantation Hospital Universitario Puerta de Hierro-Majadahonda Madrid Spain madrid.org; ^4^ Surgery Department Medical School IDIPHISA Universidad Autónoma de Madrid (UAM) Madrid Spain uam.es; ^5^ Department of Anesthesiology and Resuscitology Okayama University Graduate School of Medicine, Dentistry, and Pharmaceutical Sciences Okayama Japan okayama-u.ac.jp

**Keywords:** chronic lung allograft dysfunction, perfusion scintigraphy, single lung transplantation

## Abstract

**Background:**

The physiological relevance of postoperative perfusion distribution after single lung transplantation (SLT) remains poorly defined, as asymmetric blood flow toward the graft is often regarded as an inherent consequence of the remaining native lung. This study aimed to determine whether the graft‐directed perfusion ratio assessed by quantitative lung perfusion scintigraphy (QLPS) in the early postoperative period serves as an early physiological marker after SLT.

**Methods:**

We retrospectively analyzed SLT recipients who underwent surgery at Okayama University Hospital between 1998 and 2021 and completed QLPS approximately 3 months posttransplant. The graft perfusion ratio (graft flow/total pulmonary flow × 100%) was quantified, and recipients were classified into high (≥ 80%) and low (< 80%) perfusion groups. CLAD‐free survival, overall survival, and longitudinal perfusion changes were compared using Kaplan–Meier analysis and repeated‐measures ANOVA.

**Results:**

Recipients with higher graft perfusion demonstrated significantly better CLAD‐free survival (*p* = 0.027). Overall survival was higher in the high‐perfusion group, although the difference did not reach statistical significance (*p* = 0.075). Repeated‐measures ANOVA revealed a significant group‐by‐time interaction (*p* = 0.019): recipients who developed CLAD had lower graft perfusion as early as 3 months after transplantation, followed by a progressive decline, whereas those who did not develop CLAD maintained stable perfusion. In the multivariable Cox analysis, higher graft perfusion was associated with a lower hazard of reaching the composite CLAD‐free survival endpoint.

**Conclusions:**

Early postoperative graft perfusion assessed by QLPS may serve as a noninvasive physiological marker after SLT. Higher graft‐directed perfusion was associated with a lower hazard of reaching the composite CLAD‐free survival endpoint and may aid early risk stratification and posttransplant surveillance.

## 1. Introduction

Lung transplantation remains a life‐saving intervention for patients with end‐stage lung diseases refractory to medical therapy. In bilateral lung transplantation (BLT), both lungs are procured from the same donor and implanted simultaneously, thereby establishing relatively symmetric graft perfusion and ventilation. Recent evidence has demonstrated that when a significant intergraft perfusion differential arises, this asymmetry correlates with adverse outcomes, including earlier onset of chronic lung allograft dysfunction (CLAD) and reduced overall survival [1]. Such asymmetries are thought to represent localized vascular injury, bronchial complications, or differential immunologic responses between grafts, suggesting that post‐BLT perfusion asymmetry may serve as an early physiological marker of unilateral graft compromise.

In contrast, single lung transplantation (SLT) constitutes a distinct physiological state in which the native lung remains in situ. This configuration inherently produces substantial perfusion asymmetry between the transplanted and native lungs. Redistribution of blood flow toward the allograft is often interpreted as favorable, reflecting functional integration of the transplanted lung. Nevertheless, the clinical implications of such postoperative perfusion asymmetry remain poorly defined. It is uncertain whether increased graft perfusion simply mirrors the severity of native lung dysfunction or instead reflects preserved vascular integrity and favorable graft adaptation.

Despite the global predominance of BLT, SLT continues to hold significant clinical value for carefully selected recipients. Analyses of large international registries indicate that while crude survival rates tend to favor BLT, this advantage attenuates after adjustment for recipient age, underlying disease, and preoperative severity. Among older patients and those with idiopathic pulmonary fibrosis, SLT confers a clear survival benefit compared with continued waiting, underscoring its ongoing therapeutic relevance [2]. Furthermore, given the persistent global donor shortage, SLT remains a pragmatic and ethically important approach. However, recent data show that the contralateral donor lung is frequently discarded when only one lung is transplanted, highlighting the need for improved donor–recipient matching and allocation strategies to maximize organ utilization [3].

Prior investigations have predominantly focused on pretransplant quantitative lung perfusion scans (QLPS) to assist in determining which lung should be replaced. Fox et al. demonstrated that transplantation of the less‐perfused (“side‐mismatched”) lung did not adversely influence postoperative outcomes, implying that preoperative perfusion asymmetry alone does not dictate prognosis [4]. By contrast, little is known regarding posttransplant perfusion redistribution, which reflects the dynamic physiological interaction between the native and transplanted lungs. In BLT, intergraft perfusion imbalance has been associated with early CLAD development and diminished survival, suggesting that hemodynamic symmetry contributes to long‐term graft stability. Whether analogous mechanisms apply to SLT—where graft and native lung differ markedly in compliance and vascular resistance—remains undetermined.

Accordingly, we performed a retrospective analysis of postoperative perfusion distribution in a well‐characterized cohort of SLT recipients to examine its relationship with long‐term outcomes. We hypothesized that the proportion of total pulmonary perfusion directed toward the transplanted lung would provide an integrated measure of posttransplant pulmonary hemodynamics and might serve as an early physiological correlate of long‐term outcomes.

## 2. Materials and Methods

### 2.1. Study Design and Population

This retrospective single‐center study screened all 135 deceased‐donor lung transplant recipients treated at Okayama University Hospital between October 1998 and December 2021. Of these, 46 underwent SLT. Two recipients without postoperative quantitative lung perfusion scintigraphy (QLPS) performed approximately 3 months after transplantation were excluded, leaving 44 recipients for the final analysis. The study was approved by the Institutional Review Board of Okayama University Hospital (approval number 2510‐034), and the requirement for informed consent was waived because of its retrospective nature.

### 2.2. Assessment of Postoperative Lung Perfusion

Quantitative lung perfusion scintigraphy was routinely performed before transplantation as part of the pretransplant evaluation to assess the relative perfusion of each lung. Postoperative pulmonary perfusion was evaluated using ventilation–perfusion scintigraphy (V/Q scan) performed at approximately 3 months after SLT, when early postoperative recovery was considered stable and graft perfusion had reached a steady state. This timing was deliberately selected to minimize confounding effects from acute perioperative events, such as primary graft dysfunction (PGD), and to assess the sustained physiological adaptation of the allograft. The proportion of perfusion directed to the transplanted lung was expressed as a percentage of total bilateral pulmonary blood flow. Because perfusion was expressed as the relative contribution of each lung to total pulmonary perfusion, native lung perfusion was mathematically complementary to graft perfusion (100 − graft perfusion). To explore the prognostic impact of graft perfusion distribution, both continuous‐variable and threshold‐based approaches were employed. The continuous percentage of graft perfusion was analyzed as a continuous exposure in relation to the composite CLAD‐free survival endpoint. For secondary visualization, patients were additionally stratified into two groups using a pragmatic cutoff of 80% perfusion, which approximated the cohort median (77%). Patients were additionally stratified using a pragmatic cutoff of 80% graft perfusion, which approximated the cohort median (77%) and was consistent with the inflection region identified in the restricted cubic spline analysis. This threshold was used solely for visualization and descriptive comparisons, whereas the primary analyses treated graft perfusion as a continuous variable.

### 2.3. Longitudinal and Comparative Analyses

To investigate the temporal dynamics of graft perfusion, serial V/Q scans (when available) were analyzed to compare changes in the perfusion ratio between patients who subsequently developed CLAD and those who did not. A two‐way repeated‐measures analysis of variance (ANOVA) was performed with time (within‐subject factor) and CLAD status (between‐subject factor) as fixed effects to assess whether perfusion trajectories differed between groups. In addition, the pretransplant perfusion ratio of the lung later selected for transplantation was compared with the corresponding posttransplant graft perfusion to assess the extent of perfusion redistribution induced by transplantation.

### 2.4. Outcome Measures

The primary outcome was CLAD‐free survival, defined as the time from transplantation to the onset of CLAD, retransplantation, or death, whichever occurred first. Overall survival was analyzed as a secondary endpoint.

### 2.5. Statistical Analysis

Continuous variables are presented as medians with ranges, and categorical variables as counts and percentages. Between‐group comparisons were performed using the Mann–Whitney *U* test or chi‐square test, as appropriate. Survival curves were estimated using the Kaplan–Meier method, and differences were compared using the log‐rank test. A multivariable Cox proportional hazards regression model was constructed to assess associations with the composite CLAD‐free survival endpoint. Given the limited number of CLAD events, only two clinically relevant covariates were included to avoid model overfitting. Accordingly, adjustment for all baseline variables, including underlying diagnosis, was not feasible. Residual confounding cannot be excluded. The primary explanatory variable was the postoperative graft perfusion ratio (continuous variable, per 10%‐point increase), whereas mismatch transplantation (categorical: mismatch vs. match) was included as an adjustment covariate because of its potential influence on postoperative perfusion distribution. The proportional hazards assumption was verified graphically and statistically. To examine the robustness of findings, sensitivity analyses were conducted using alternative dichotomization thresholds of 75% and 85%.

Furthermore, a restricted cubic spline model with three knots (at the 10th, 50th, and 90th percentiles) was applied to flexibly evaluate the continuous relationship between graft perfusion and the hazard of reaching the composite CLAD‐free survival endpoint.

All analyses were performed using JMP Pro Version 18 (SAS Institute Inc., Cary, NC, USA) and R Version 4.3.0 (R Foundation for Statistical Computing, Vienna, Austria). A two‐sided *p* value < 0.05 was considered statistically significant.

## 3. Results

### 3.1. Patient Characteristics

A total of 44 recipients of SLT from brain‐dead donors who underwent QLPS at 3 months posttransplantation were analyzed. Baseline characteristics of the study population are summarized in Table 1. Recipients were stratified according to graft perfusion ratio at 3 months (≥ 80% vs. < 80%). The high‐perfusion group exhibited a lower incidence of acute rejection within the first posttransplant year compared with the low‐perfusion group. Early postoperative pulmonary function measured at approximately 3 months after transplantation was available in a subset of recipients. No significant differences were observed between the two groups in FEV_1_ (1.53 vs. 1.48 L, *p* = 0.717) or FVC (1.95 vs. 1.99 L, *p* = 0.865) (Table 1). CLAD phenotypes also differed between the perfusion groups (*p* = 0.02). BOS occurred in 8 recipients (33%) in the < 80% perfusion group and 1 recipient (5%) in the ≥ 80% group, whereas RAS occurred in 2 recipients (8%) and no recipients, respectively (Table 1).

**TABLE 1:** Recipient variables.

Variables	Total *n* = 44	Transplanted lung perfusion < 80% *n* = 24	Transplanted lung perfusion ≥ 80% *n* = 20	*p* value
Age (years), median (range)	52 (42–58)	52 (46.5–57)	48 (40–59)	0.93
Sex				0.96
Male	27 (61%)	15 (63%)	12 (60%)	
Female	17 (39%)	9 (37%)	8 (40%)	
BMI, kg/m^2^, median (range)	20.6 (18.1–25.3)	22.8 (19.1–26.1)	19.0 (18.2–21.5)	0.13
Indication, *n* (%)				0.41
ILD	20 (46%)	13 (54%)	7 (35%)	
GVHD	5 (11%)	3 (8%)	2 (10%)	
COPD	8 (18%)	3 (8%)	5 (25%)	
Other	11 (25%)	5 (21%)	6 (30%)	
LAS, median (range)	34.4 (33.2–38.6)	34.4 (33.2–39.1)	34.1 (32.7–36.4)	0.37
Pretransplant medical condition				0.45
ECMO, *n* (%)	0 (0%)	0 (0%)	0 (0%)	
Invasive mechanical ventilation, *n* (%)	7 (16%)	3 (8%)	4 (20%)	
Noninvasive mechanical ventilation, *n* (%)	37 (84%)	21 (288%)	15 (75%)	
Intraoperative CPB, *n* (%)	9 (20%)	4 (17%)	5 (25%)	0.43
PGD Grade 2, 3 (within 72 h), *n* (%)	19 (43%)	9 (37%)	10 (20%)	0.4
PGD Grade 3 (within 72 h), *n* (%)	11 (25%)	6 (25%)	5 (25%)	1
Acute cellular rejection	15 (34%)	12 (50%)	3 (16%)	0.019
Acute mediate rejection	6 (14%)	5 (21%)	1 (5%)	0.14

*Note:* Data are presented as median (range) or *n* (%). ECMO: extracorporeal membrane oxygenation. CPB: cardiopulmonary bypass.

Abbreviations: BMI, body mass index; CLAD, chronic lung allograft dysfunction; COPD, chronic obstructive pulmonary disease; GVHD, graft versus host disease; ILD, interstitial lung disease; LAS, lung allocation score; MV, mechanical ventilation; PH, pulmonary hypertension; PGD, primary graft dysfunction.

### 3.2. Association Between Postoperative Perfusion and CLAD‐Free Survival

In the multivariable Cox regression model, mismatch transplantation was not significantly associated with CLAD‐free survival (HR: 1.02; 95% CI: 0.26–4.00; *p* = 0.97). In contrast, a higher postoperative perfusion ratio of the transplanted lung was associated with a lower hazard of reaching the composite CLAD‐free survival endpoint (HR: 0.62 per 10‐percentage‐point increase; 95% CI: 0.39–0.99; *p* = 0.045) (Table 2).

**TABLE 2:** Multivariable Cox proportional hazards analysis for CLAD.

Variables	Hazard ratio	95% confidence interval	*p* value
Transplanted lung perfusion (%) (per 10% increase)	0.62	0.39–0.99	0.045
Mismatch lung transplantation (yes vs. no)	1.02	0.26–4.00	0.97

Abbreviation: CLAD, chronic lung allograft dysfunction.

### 3.3. Threshold‐Based Visualization and Survival Comparison

To facilitate clinical interpretation, the continuous association was visualized using an 80% perfusion threshold. As shown in Figure 1, recipients with graft perfusion ≥ 80% demonstrated significantly better CLAD‐free survival compared with those with < 80% perfusion (log‐rank *p* = 0.027). When overall survival was analyzed according to the same threshold (Figure 2), patients in the high‐perfusion group demonstrated higher long‐term survival, although the difference did not reach statistical significance (*p* = 0.075).

**FIGURE 1:**
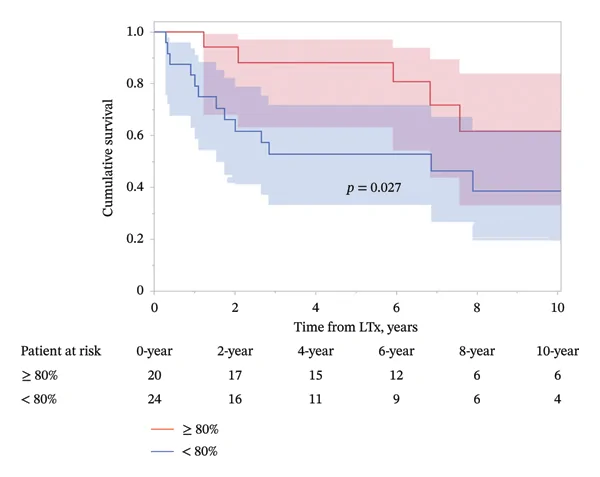
CLAD‐free survival according to postoperative graft perfusion. Kaplan–Meier curves comparing CLAD‐free survival between recipients with high (≥ 80%) and low (< 80%) graft perfusion ratios at 3 months after single lung transplantation. Patients with higher graft‐directed perfusion exhibited significantly better CLAD‐free survival (log‐rank *p* = 0.027).

**FIGURE 2:**
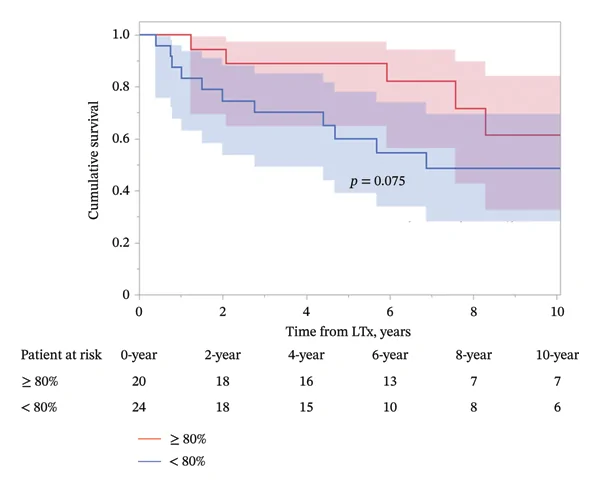
Overall survival according to postoperative graft perfusion. Kaplan–Meier curves comparing overall survival between high (≥ 80%) and low (< 80%) perfusion groups. The high‐perfusion group demonstrated higher overall survival, although the difference did not reach statistical significance (log‐rank *p* = 0.075).

### 3.4. Sensitivity and Spline Analyses

In sensitivity analyses, alternative thresholds of 75% and 85% yielded results in the same direction (Table 3). Restricted cubic spline modeling showed that the hazard of reaching the composite CLAD‐free survival endpoint decreased with increasing graft perfusion and plateaued beyond approximately 75%–80% (Figure S1). This pattern supported the use of 80% as a convenient threshold for visualization, while the association appeared continuous rather than dichotomous. The variation in statistical significance across thresholds likely reflects the limited sample size and number of events rather than evidence of a discrete biological threshold.

**TABLE 3:** Sensitivity analyses of postoperative graft perfusion thresholds for CLAD‐free survival.

Threshold of transplanted lung perfusion (%)	HR (high ≥ threshold vs. low < threshold)	95% CI (lower–upper)	*p* value
75	0.65	0.41–1.02	0.062
80	0.58	0.36–0.94	0.028
85	0.61	0.37–1.05	0.073

Abbreviations: CI, confidence interval; CLAD, chronic lung allograft dysfunction; HR, hazard ratio.

### 3.5. Longitudinal Changes in Graft Perfusion and CLAD Development

Serial perfusion data revealed distinct temporal patterns between patients who did and did not develop CLAD (Figure 3). Repeated‐measures ANOVA demonstrated a significant group × time interaction (*p* = 0.019), indicating that the change in perfusion over time differed significantly between the two groups. Specifically, graft perfusion in the CLAD group was already lower at 3 months posttransplant and declined further during follow‐up, whereas perfusion in the non‐CLAD group remained stable across all observation points. These findings demonstrate divergent longitudinal perfusion trajectories between recipients who subsequently developed CLAD and those who did not.

**FIGURE 3:**
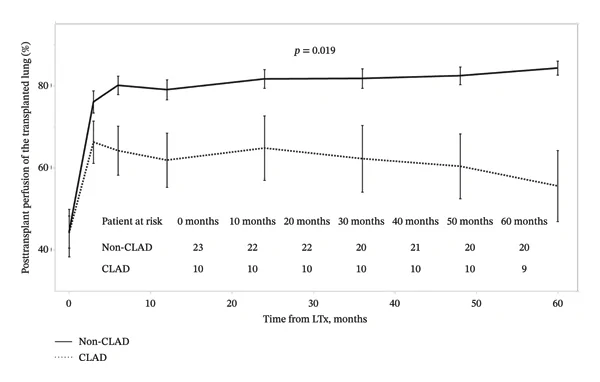
Longitudinal changes in graft perfusion according to CLAD development. Temporal changes in graft perfusion ratios were compared between recipients who developed CLAD and those who did not. Repeated‐measures ANOVA revealed a significant group × time interaction (*p* = 0.019), indicating divergent perfusion trajectories between groups. In the CLAD group, graft perfusion was already lower at 3 months and declined further during follow‐up, whereas perfusion in the non‐CLAD group remained stable. The number of recipients with available QLPS in the non‐CLAD/CLAD groups at baseline and 10, 20, 30, 40, 50, and 60 months was 23/10, 22/10, 22/10, 20/10, 21/10, 20/10, and 20/9, respectively, as shown below the plot.

A scatter plot comparing the pretransplant perfusion of the lung selected for transplantation with its posttransplant perfusion is shown in Figure 4. Most recipients exhibited an increase in graft perfusion after transplantation, reflecting hemodynamic redistribution toward the graft. In contrast, three recipients demonstrated paradoxically reduced postoperative perfusion compared with their pretransplant values; notably, all three had postoperative graft perfusion below 80% at 3 months, consistent with the high‐risk category identified in our main analysis. All three of these recipients subsequently developed CLAD, and in two of them, CLAD occurred within 2 years after transplantation. Both of these patients died within 5 years posttransplantation. These cases illustrate that failure to achieve the usual postoperative redistribution toward the graft may identify an unfavorable posttransplant physiological state. However, the relative contributions of graft dysfunction and persistent native‐lung perfusion cannot be determined from the present data.

**FIGURE 4:**
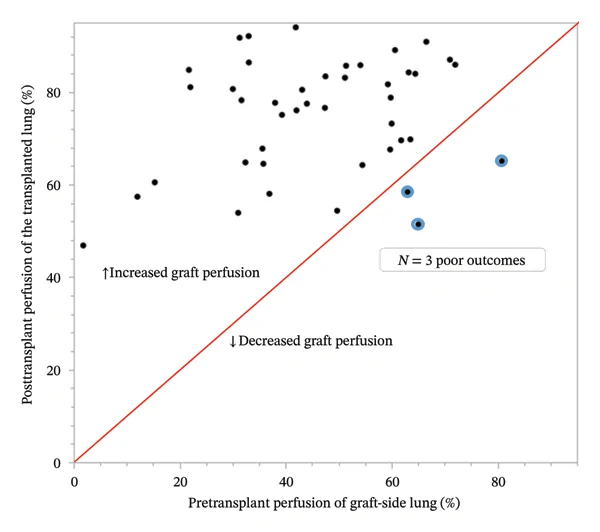
Relationship between pre‐ and posttransplant perfusion of the transplanted lung. A scatter plot comparing the pretransplant perfusion of the lung selected for transplantation with its posttransplant perfusion at 3 months. Most cases exhibited an increase in graft perfusion following transplantation; however, three recipients demonstrated a paradoxical reduction (all < 80%), with postoperative perfusion lower than the pretransplant value (below the identity line). All three recipients subsequently developed CLAD, two within 2 years after transplantation, and both of these patients died within 5 years.

## 4. Discussion

The present study suggests that postoperative graft perfusion, quantified by QLPS approximately 3 months after SLT, may be associated with subsequent CLAD‐free survival. Recipients with graft perfusion below 80% had poorer CLAD‐free survival, and higher graft perfusion was associated with a lower hazard of reaching the composite CLAD‐free survival endpoint in the multivariable analysis. Because causality cannot be inferred and QLPS expresses relative bilateral perfusion, postoperative perfusion distribution should be interpreted as an integrated physiological marker of the interaction between the transplanted and native lungs, rather than as a graft‐specific measure.

After SLT, the transplanted and native lungs coexist within the same cardiopulmonary circuit yet differ markedly in compliance, pulmonary vascular resistance, and ventilatory mechanics. Blood‐flow distribution may therefore be influenced by graft and native‐lung factors, including vascular resistance, regional compliance, ventilation, and progression of native‐lung disease. A lower graft perfusion ratio may reflect several possible physiological states rather than a single graft‐specific process. Perfusion distribution is thus best interpreted as a quantitative indicator of the dynamic interaction between the transplanted and native lungs, not as evidence of a particular mechanism.

Our results extend previous observations from BLT, where intergraft perfusion imbalance has been associated with earlier CLAD onset and inferior survival [1, 5]. In BLT, asymmetry is interpreted as a manifestation of unilateral vascular injury, airway complications, or differential immune activation. The present findings suggest that a similar physiological principle may apply to SLT, despite its distinct hemodynamic environment. In the single‐lung setting, blood flow distribution is determined by the balance between graft compliance and native‐lung resistance, making perfusion asymmetry an indicator of the overall integration of the graft into the cardiopulmonary system. Importantly, pretransplant perfusion asymmetry (“side‐mismatch”) has not been shown to influence postoperative outcomes [4], and our data reaffirm that postoperative redistribution—reflecting a new hemodynamic equilibrium rather than baseline anatomy—may offer a more informative perspective.

A key interpretive issue is whether postoperative perfusion predominantly reflects intrinsic graft physiology, persistent native‐lung perfusion, or their interaction. Because QLPS expresses the relative contribution of each lung to total pulmonary perfusion, the present data cannot separate these components. Moreover, early allograft injury may influence the 3‐month perfusion distribution. Postoperative graft‐directed perfusion should therefore be interpreted as an integrated physiological marker rather than a graft‐specific measure, and the underlying mechanisms require further investigation.

Previous studies have provided mixed results regarding the prognostic significance of early perfusion imaging. Belmaati et al. reported that ventilation–perfusion scintigraphy performed 3 months after SLT did not correlate with early PGD or subsequent changes in lung function within the first year, suggesting that early PGD does not influence later perfusion patterns [6]. In contrast, Hardoff et al. demonstrated that reduced relative perfusion of the transplanted lung within 1–3 months predicted subsequent chronic rejection (bronchiolitis obliterans), although their findings were derived from a small cohort and unadjusted analyses with variable assessment timing. The present study builds on these observations by evaluating a larger and more contemporary cohort using a uniform 3‐month QLPS assessment and long‐term follow‐up, thereby providing a more refined understanding of the prognostic value of postoperative perfusion [7].

The longitudinal divergence in perfusion trajectories is consistent with an association between early perfusion distribution and subsequent outcomes, but it does not establish the underlying mechanism. Three recipients exhibited paradoxically reduced graft perfusion after transplantation (Figure 4), and all subsequently developed CLAD. Two of these recipients experienced Grade 3 PGD, suggesting that early allograft injury may have contributed to impaired redistribution. Pretransplant mPAP was available in only one of these recipients, precluding meaningful assessment of pulmonary hypertension. Early postoperative lung function is an important physiological parameter after SLT, as recently demonstrated by Gerckens et al. [8]. In our cohort, however, early postoperative FEV_1_ and FVC did not differ significantly between the < 80% and ≥ 80% perfusion groups. Thus, the respective contributions of graft injury, native‐lung physiology, pulmonary vascular factors, and subsequent CLAD cannot be determined from this small cohort.

The early divergence in perfusion trajectories suggests that QLPS may provide physiological information before measurable changes in spirometry or imaging become evident. However, these observations do not establish that altered perfusion reflects intrinsic graft dysfunction, and the mechanisms underlying the association remain uncertain [9–11].

From this perspective, the graft‐directed perfusion ratio should be interpreted as a noninvasive physiological marker associated with outcome, not as a causal determinant or graft‐specific measure of injury. Further studies integrating perfusion data with endothelial, molecular, vascular, and native‐lung assessments are needed to clarify the mechanisms underlying this association.

The comparison with BLT physiology also helps contextualize the present findings. In BLT, perfusion distribution is not perfectly symmetrical; mild right‐sided predominance is considered physiological. Li et al. reported mean relative perfusion values of approximately 45 ± 8% to the left lung and 55 ± 8% to the right lung after brain‐death BLT, and differences of up to approximately 10% were common and were not associated with adverse outcomes 1. In SLT, even a graft share of 70%–80% may represent a higher absolute flow than that received by either lung after BLT. Therefore, our results should not be interpreted as defining a pathological threshold in absolute terms. The 80% threshold was selected as a pragmatic, cohort‐based cutoff for visualization because it approximated the cohort median and corresponded to the region where the spline curve began to plateau; the association appeared continuous, without a distinct boundary.

Clinically, a lower graft perfusion ratio at 3 months may identify recipients who warrant closer surveillance. Our findings may also have implications for BLT, in which postoperative perfusion asymmetry has been associated with adverse long‐term outcomes, although the interpretation of asymmetry differs between SLT and BLT. Serial QLPS may provide additional information on temporal changes in perfusion; however, its clinical benefit remains uncertain in the absence of an established intervention targeting abnormal perfusion patterns. Selective reassessment may therefore be more appropriate in recipients with abnormal early perfusion or subsequent clinical deterioration. Because QLPS requires nuclear medicine resources, additional patient time, and cost, its incremental value and cost‐effectiveness relative to routinely performed imaging modalities such as CT require further evaluation.

Several limitations should be acknowledged. First, this was a retrospective, single‐center study with a relatively small sample size, limiting statistical power, subgroup analyses, and the complexity of multivariable modeling. Although no statistically significant differences in baseline diagnostic indications were observed between the perfusion groups, clinically relevant differences in underlying disease composition may have remained undetected because of the limited sample size. Consequently, residual confounding related to recipient diagnosis cannot be excluded. The timing of perfusion assessment at 3 months was chosen to minimize acute confounders, but prospective validation with serial imaging is required to confirm the temporal stability of perfusion‐based risk stratification. Potential confounding by unmeasured factors—such as perioperative hemodynamics, ventilatory settings, residual disease progression in the native lung, and early acute cellular rejection—cannot be excluded. Because all episodes of acute cellular rejection occurred before the 3‐month perfusion assessment, early allograft injury may have influenced postoperative graft perfusion and therefore may partly account for the observed association with subsequent CLAD‐free survival. Accordingly, reverse causation cannot be completely excluded. Nonetheless, the consistent associations observed across continuous, categorical, and spline analyses, and their persistence across disease categories, lend support to the overall reliability of these findings. Furthermore, the graft perfusion ratio is an inherently relative measurement and does not allow the respective contributions of the transplanted and native lungs to be quantified separately. Changes in native‐lung disease, vascular resistance, ventilation, and compliance may influence the observed perfusion distribution. In addition, pulmonary artery caliber and anastomotic morphology were not systematically quantified on postoperative CT; therefore, anatomical vascular factors contributing to differences in graft perfusion cannot be excluded. Therefore, the present findings should not be interpreted as demonstrating that low graft‐directed perfusion specifically represents intrinsic graft dysfunction. Studies combining perfusion scintigraphy with lung‐specific physiological measurements, ventilation imaging, pulmonary vascular assessment, and quantitative imaging of the native lung are needed to clarify the underlying mechanisms.

In summary, postoperative perfusion distribution assessed by QLPS may serve as an early physiological marker after SLT. Higher graft‐directed perfusion was associated with a lower hazard of reaching the composite CLAD‐free survival endpoint. Because QLPS is a relative measure, this association should not be interpreted as evidence of a graft‐specific mechanism or a causal effect of perfusion. QLPS may nevertheless provide a quantitative metric for risk stratification and individualized surveillance, subject to prospective validation.

## Funding

No funding was received for this manuscript.

## Ethics Statement

This study was approved by the Institutional Review Board of Okayama University Hospital (approval no. 2510‐034).

## Conflicts of Interest

The authors declare no conflicts of interest.

## Supporting Information

Additional supporting information can be found online in the Supporting Information section.

## Supporting information


**Supporting Information** Figure S1. Restricted cubic spline analysis of graft perfusion and CLAD risk. Restricted cubic spline modeling of the continuous association between postoperative graft perfusion and the hazard of CLAD. The adjusted hazard of CLAD generally decreased with increasing graft perfusion and approached a plateau at approximately 80%, providing a rationale for the use of 80% as a practical cutoff in this cohort. The solid line represents the adjusted hazard ratio, and the shaded area represents the 95% confidence interval. The median graft perfusion (77%) was used as the reference.

## Data Availability

The data that support the findings of this study are available from the corresponding author upon reasonable request.
